# Neurosurgery Program Directors’ Perspectives on the Role of Research in Residency Matching: A Qualitative Study

**DOI:** 10.7759/cureus.89823

**Published:** 2025-08-11

**Authors:** Garrett Dyess, Danner Butler, David Williams, Maxon Bassett, Michael Rallo, Adnan Shahid, Mehdi Khaleghi, Matthew Tao, Jai Thakur, Lola Chambless

**Affiliations:** 1 Medical School, University of South Alabama College of Medicine, Mobile, USA; 2 Neurological Surgery, Rutgers Robert Wood Johnson Medical School, New Brunswick, USA; 3 Neurological Surgery, University of South Alabama College of Medicine, Mobile, USA; 4 Neurological Surgery, Vanderbilt University Medical Center, Nashville, USA

**Keywords:** match, medical student research, program directors, qualitative analysis, residency match

## Abstract

Background and objectives: Given growing concerns about an “arms race” among medical students to increase research output, it is essential to examine the role of research in the neurosurgery selection process by analyzing the diverse perspectives of program directors (PDs) nationwide.

Methods: All neurosurgery PDs were contacted to participate in structured interviews with purposive criterion-based sampling and thematic analysis. Those who agreed were asked eight open-ended questions regarding the role of medical student research in residency selection. Their responses were analyzed using qualitative research techniques.

Results: A total of 31 (26.31%) PDs agreed to participate in interviews. Overall, research was viewed as a marker of interest and dedication to neurosurgery, with a focus on evaluating the quality of the journal the work was published, the authorship status of the student, and the ability to explain research. Most PDs interviewed value research experiences they determine to be of high quality over the pure quantity of research produced, but do not favor either basic science or clinical-based research. PDs also reported accounting for access to research support staff, infrastructure, and opportunity when assessing the research experience of applicants. Many PDs are concerned with the previously mentioned medical student research “arms race,” voicing concern over a decrease in research quality as a potential long-term result. However, others believe that the increasing quantity of papers can be attributed to the competitive and demanding nature of neurosurgery.

Conclusion: While neurosurgery PDs value medical student research as a metric for interest and dedication to the field, opinions vary regarding the “arms race” of research as the number of publications per applicant continues to steadily increase, prompting discussions regarding potential solutions to preserve the quality of academic research moving forward.

## Introduction

Medical students are publishing more research than ever before [1]. Nearly all specialties have seen a surge in the number of abstracts, presentations, and publications reported through the Electronic Residency Application Service (ERAS) and collected by the National Residency Matching Program (NRMP) [2,3]. Notably, neurosurgery topped the list in 2024 with an average of 37.4 items per applicant. Many traditionally competitive specialties also show steeper increases in research activity compared to less competitive fields [3]. 

There are growing concerns about medical student research. Some have described this trend as an “arms race,” resulting in a “publish or perish” culture [1]. However, diverse viewpoints exist among program leadership on how to address the issue. Various policy decisions have been discussed, ranging from further limiting the number of research entries applicants can report through ERAS to standardizing assessments of medical students' research productivity relative to the institutions from which they graduated [4-6]. The debate concerning the proper path forward continues, emphasizing the need for well-contextualized and comprehensive research into the issue.

Given the complexity of this ongoing discussion, the authors believed that studying the role of research in the neurosurgery residency selection process using a qualitative approach to capture the variability and nuance of program director (PD) viewpoints was warranted.

Qualitative research methodologies have been utilized to address multifactorial phenomena that may not be adequately captured by traditional surveys or Likert scales. These methodologies are well-suited for exploring the "how," "what," or "why" of the phenomena in question [7-9].

We chose a qualitative approach to build upon previous quantitative research on the topic. While the importance of research in the neurosurgery match is well-documented, the authors identified important nuances that remained unaddressed and have provided direct quotations from PDs [3-6]. These nuances became the objectives of our study: to understand how neurosurgery PDs evaluate medical student research, specifically, the balance between quality and quantity, preferences for basic science versus clinical research, the role of research before and after interviews, and how access to research opportunities is factored into their assessment. Through structured interviews, we also explored their views on the broader purpose of student research, its impact on selection decisions, and perspectives on the growing research 'arms race' among applicants.

## Materials and methods

Rationale

Medical education research increasingly incorporates qualitative, quantitative, and mixed-methods approaches to address complex questions. Although quantitative methods are traditionally rooted in a positivist paradigm that emphasizes objective measurement, this framework may not fully capture the nuance of certain experiences, such as the neurosurgery residency match process, necessitating alternative methodologies [7].

Qualitative research has gained visibility in major medical journals and has been applied to topics such as gender disparities in surgery, residency attrition, compensation models, and implementation barriers for enhanced recovery protocols [8-11]. Major advancements in computational power have significantly enhanced our ability to analyze large datasets, most notably through Natural Language Processing (NLP) and specialized software such as Qualitative Data Analysis (QDA) Miner (Provalis Research, Montreal, Canada). In designing this study, the authors followed methodological guidance from JAMA Surgery’s Practical Guide to Qualitative Research [12] and adhered to the Standards for Reporting Qualitative Research (SRQR) checklist, a qualitative reporting standard analogous to Preferred Reporting Items for Systematic Reviews and Meta-Analyses (PRISMA) and Consolidated Standards of Reporting Trials (CONSORT).

Study design

The study was conducted at the University of South Alabama in Mobile, Alabama, United States. A purposive criterion-based sampling technique was utilized to identify neurosurgery PDs across the United States. The American Association of Neurological Surgery (AANS) maintains a neurosurgery residency training program directory, which was utilized to build our cohort [13]. Eligible participants were contacted through email and asked to participate in a structured interview at a neurosurgical conference, over the phone, or on Zoom (Zoom Communications, Inc., CA, USA). If candidates did not respond, no further attempts at contact were made. Informed consent, ensuring anonymity of participant responses, was obtained prior to the first question of each interview. No financial compensation resulted from the interview. The study was approved by the Institutional Review Board at the University of South Alabama (IRB Number: 23-337/2086707-1).

A structured interview consisting of eight open-ended questions based on discussion between three authors (GAD, MSR, LBC) was developed (Appendix 1). The research team designed these questions to address five key domains deemed most relevant to applicants who would be consulting this article. These domains included the impact of research on securing interview invitations, its role in final residency selection, preferences for basic science versus clinical research, fairness in access to research opportunities across institutions, and preferences for quality versus quantity of publications.

The interview template was strictly followed, leading to no follow-up questions, and questions were not changed during the interview phase of the project. Interviews were conducted between February 17, 2024, and March 15, 2024. Interviews were recorded and stored on a secure device and transcribed using Zoom’s transcription service. Field notes were collected from each interviewer upon completion of the last interview.

Research team

Our research team consisted of four medical students at varying stages of their education, all interested in pursuing neurosurgery (GAD, DWB, MLB, MSR). One member is an educational researcher with expertise in qualitative analysis (DSW). The two attending neurosurgeons (JDT and LBC) on the team included a PD who was also an interview participant (LBC). 

Coding

Training and oversight of the qualitative coding process were provided by DSW. Transcripts were inductively coded by two team members (GAD and DWB), who independently reviewed each transcript line by line. A preliminary codebook was developed through both manual review and use of QDA Miner’s cluster coding algorithm, which was employed to identify word associations and suggest potential codes; however, final code selection was determined through manual coding and consensus between the primary coders [14]. Coding discrepancies were discussed between the two coders, and in instances where consensus could not be reached, a third coder (MLB) adjudicated. This process of investigator triangulation, in which multiple coders contribute to theme development and resolution of differences, was used to enhance the credibility of findings.

The coding process was iterative. Regular meetings were held to refine the codebook, clarify definitions, and incorporate new insights as additional transcripts were analyzed. Open codes, descriptive labels applied to specific segments of text to capture discrete ideas or observations, were generated inductively from the data. Coding continued across transcripts until all interviews were reviewed. Thematic saturation was not reached, as novel concepts, though infrequent, continued to emerge throughout the coding process. After all transcripts were coded, the team conducted a comprehensive group review, reconciling code applications and finalizing the open codes for each of the eight interview questions. Additionally, Coders 1 and 2 agreed on 86% of the codes, while the remaining 14% were resolved by Coder 3.

Once open coding was complete, the team synthesized related codes into broader conceptual categories, known as axial codes. These represent higher-order themes that explain patterns and relationships among the open codes. Axial coding was conducted collaboratively by all three coders through discussion and consensus-building, aiming to link codes around central ideas or domains of meaning.

**Figure 1:**
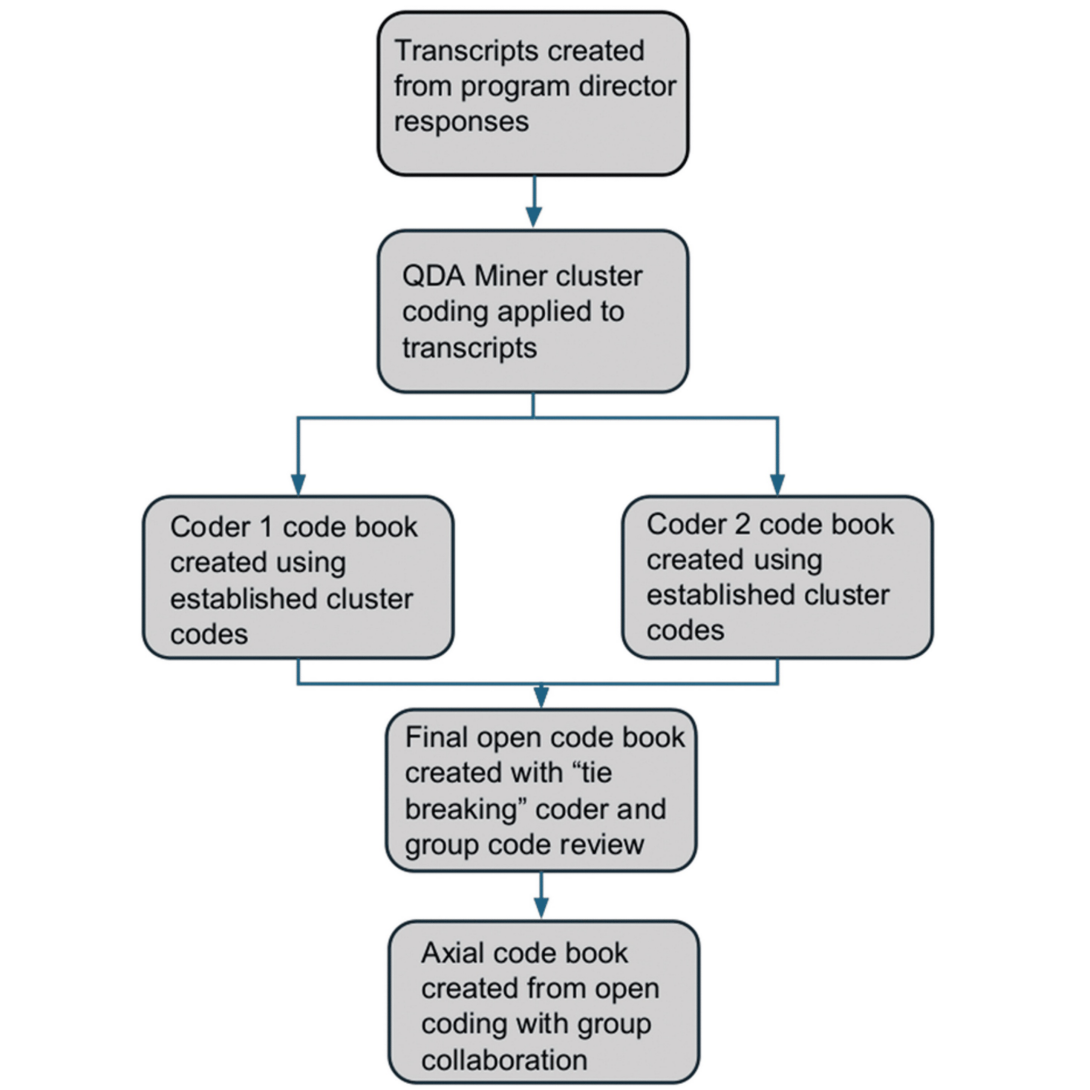
Coding workflow for qualitative analysis of program director responses Image credit: Danner Butler

All coded data were stored securely on a password-protected, cloud-based drive. Analysis was conducted using constant comparison methods to identify both convergences and divergences across interviews. Although member checking was not performed, due to the availability of direct transcripts from Zoom’s AI transcription service and time constraints for interviewees, analytic rigor was enhanced through independent coding, adjudicated resolution, and team-based consensus-building. A summary of the coding and theme development process is presented in Figure 1.

## Results

Of the 118 neurosurgery PDs contacted, 31 (26.31%) agreed to participate in interviews, which lasted an average of 13 minutes. Departments represented in this study varied by geographical region, resident class size, and National Institutes of Health (NIH) funding status. Given the study’s aim to explore perceptions of research in the neurosurgery match, it was important to include programs with differing levels of research funding and activity. NIH funding was used as a proxy for research activity, and this variation is reflected in Table 1.

**Table 1:** Characteristics of included residency programs

Program Characteristics	Counts and Percentages
Geographical Region	
Northeast	6 (19.4)
Midwest	7 (22.6)
South	13 (41.9)
West	5 (16.1)
Resident Class Size	
2 per year	18 (58.1)
3 per year	6 (19.4)
4 per year	7 (22.6)
National Institutes of Health Funding	
Top 25	11 (35.5)
26-50	5 (16.1)
51+	15 (48.4)

Medical student research in neurosurgery (purpose, priorities, and present realities) 

**Figure 2:**
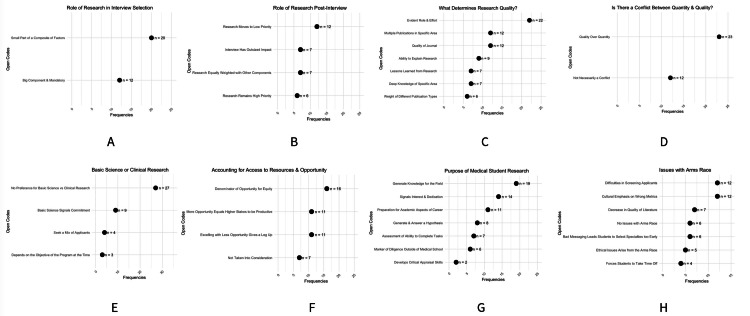
Categories of research valued by neurosurgery program directors and the influence of access on evaluation of research output A: role of research in interview selection; B: role of research post-interview; C: determinants of research quality; D: conflict between quantity and quality of research; E: preference for basic science versus clinical research; F: consideration of access to resources and opportunity; G: purpose of medical student research; H: issues related to the research “arms race.” Each dot represents the frequency (n) with which a theme was mentioned by respondents.

Questions one, two, and eight explored PDs’ perspectives on the purpose of medical student research, their preference for quantity versus quality (including how quality is assessed), and their views on the current landscape of applicant research in neurosurgery. This section presents a narrative summary of the key findings; for a comprehensive and detailed account of all responses, readers are encouraged to consult Figure 2 and Table 2. 

**Table 2:** Axial and open codes with supporting descriptions and quotations from program director interviews

Question	Axial Code/s	Open Code/s	Description/s	Supporting Quotation/s
1	Learning research methods to answer meaningful questions	Generate and answer hypotheses, generate knowledge, prepare for the academic aspects of a career, and contribute to the neurosurgical field.	An objective of medical student research is to formulate a hypothesis, design a study to test the hypothesis, and lead to meaningful results that contribute to the neurosurgery literature. These skills are then carried forward into residency and beyond.	“In my mind, the goal of that research is to develop the skill set needed to generate a hypothesis, to have an understanding of how one might investigate that hypothesis, to know the basics of data collection and management, to understand how an Institutional Review Board (IRB) functions, and when one needs to go to the IRB for permission to conduct research. Then, with assistance, perform analysis, write, and publish your work. Getting through that process as a medical student is important because it sets you up for success as a resident, where that's absolutely an expectation of every neurosurgery training program.” - Respondent #1
1	A signal of commitment and dedication of students	Assessment of ability to complete tasks, interest, and dedication, a marker of diligence, outside of the medical school curriculum.	Research experiences may indicate traits that program directors are looking for. These traits include being motivated and dedicated to neurosurgery, along with the ability to complete tasks and balance work-related tasks with research-related tasks.	“Number one, it indicates that the applicant has an interest in the field of neurosurgery that is more than superficial. So, anybody who does neurosurgical research is much more likely to have a much deeper interest in the intellectual component, and a record of publications in neurosurgical or neuroscience-related literature indicates that. They have that level of interest. The other part of it is, it's a marker for their ability to carry through a project from start to end, and kind of a marker of their academic intellectual horsepower. So, if they're unable to get a manuscript publication, that indicates a potential problem in terms of their ability to take a task from the idea stage to culmination in publication.” - Respondent #14
2	Research focus developed on a particular topic	Deep knowledge of a specific area, lessons learned from research, ability to explain research, and multiple publications in a specific area.	Research experiences are assessed by the applicants’ ability to discuss their experiences in an engaging and in-depth manner, which often occurs when applicants have explored a particular research interest through a series of research projects.	“You have students who go through four years of medical school and still find time to have 15 or 20 publications, and maybe the publications won't be in Science or Cell, but they're still reasonably good clinical publications, and that's valuable. You will have MD-PhDs who may have one paper, but it's in Nature Reports or an extremely high-impact single paper that they spent three or four years of their PhD time on. That single paper is again evidence of dedication, focus, drive, and an ability to succeed and bring a project to fruition. I also ask applicants who have multiple papers to tell me about the research effort they're most excited about or the proudest of.” - Respondent #7
2	Publication impact and contribution	Evident role and effort, weight of different publication types, and quality of the journal.	The most important aspect of research projects is assessing the amount of effort an applicant expounded. Factors which indicate effort include authorship position, more rigorous study designs, and the quality of the journal the work was accepted.	“What I look at are whether they are the first author or the last author, where it was published, and I look for the topics to see if they seem relatively robust or if they're seemingly thinner in their quality.” - Respondent #16
3	Quality is favored over quantity	Quality over quantity.	The quality of research experiences is weighed more heavily than the quantity of research experiences.	“I see people come with 50 publications, and none of them have any impact. I'm not talking about impact factor. I'm talking about impact in anything. They don't even know the importance of that. And, you know, some of the most important PhD folks that I know have few publications, but they're all important. So, I think it's critical to understand the difference between quantity and quality. And for most people, I'm not saying for all, but for most, the two don't go together. In fact, they are opposites because there's just not enough time in this world to do it.” - Respondent #12
3	Quantity and quality can co-exist, and neither is favored	Not necessarily a conflict between quantity and quality	Quantity and quality are not necessarily at odds with one another. When research is done correctly, the two may co-exist and signal an applicant's horsepower and ability to produce.	“It is an intriguing dilemma. While it's true that an extensive list of publications can sometimes dilute the overall quality of a student's work, we cannot dismiss the value of quantity outright. It showcases a student's dedication and work ethic. However, the type of publication also plays a crucial role in how we assess an applicant's contributions to the field.” - Respondent #21
4	No preference exists for basic science over clinical research	No preference for basic vs. clinical research	A subset of program directors report not preferring basic science publications over other publication types when effort is held equal.	“I think traditionally people have looked for bigger name journals, which often do cater to investigations that are scientific or bench. I am not sure that is a modern way of looking at it. We certainly don't. We don't care if it's basic or clinical science, whether it is in a neurosurgery journal or not. We really do care about the impact and the qualification of the publication. The intended impact but also the actual impact are both more important to us.” - Respondent #6
4	Basic science may indicate more commitment	Basic science can signal commitment	Basic science publications require more effort and are completed over a much longer time span than clinical publications. For this reason, some program directors weigh basic science publications more heavily than clinical publications.	“Well, I think we all understand that good basic science projects take time, lots of effort, and certainly don't generate the sheer number of publications that clinical research does, so high-quality basic science is considered at a level above clinical research. However, I do think the majority of residents these days are more involved in clinical research than basic science, so we also recognize good quality clinical research as an important predictor of their future performance.” - Respondent #19
4	Fluctuates based on the current and targeted group of trainees’ research interests	Depends on the programs' objectives at the time, seeking a mix of applicants.	A subset of program directors indicated the importance of recruiting a group of residents with diverse research interests. In instances where a specific research focus is overrepresented in the current group of residents, applicants with different research interests may be prioritized.	“We aim to have a mix in our pool of residents. We look for residents with an interest in basic science, although these are relatively few in the applicant pool. We are also happy to welcome many residents interested in clinical research, which is much more common, as well as those focused on population-based studies, socio-economic research, engineering, and similar fields. In our program, I strongly desire a mix of these interests. People with clinical retrospective research experience or clinical outcomes experience are the easiest to find. Therefore, everyone in our applicant pool tends to have some of this experience.” - Respondent #1
5	Research is a small part of a composite of factors for interview selection	A small part of a composite of many factors for interview selection.	Research is one component of many utilized to determine who is selected for an interview. In these programs, the research experiences do not have an outsized impact on interview selection.	“There's no metric per se, no algorithm that we use. We use it as a composite. So, for example, if somebody has done research that is of a very high level but hasn't done other things, that's OK. You know, we realize that this is somebody very involved. If there's somebody who has done a lot of leadership and social outreach and maybe not as much research, but they've done impactful work, absolutely, that's important. So, it's a composite of things, and I think we're willing to make a point of looking at the whole picture, so it takes more time, but I think it's important.” - Respondent #4
5	Substantial research experience is mandatory for interview selection	A big component and mandatory for selection.	Research has an outsized impact on determining who is selected for an interview at these programs. A common trait of programs that value research heavily at the interview selection phase is a robust research footprint and a goal of producing academic neurosurgeons.	“Research is one of several factors that we weigh very heavily, taken cumulatively, whether it's the quantity and/or, more recently, the quality of research experience and productivity. Generally, we see it as a metric—not necessarily the most important one, but an important one for predicting who will go into academic neurosurgery. Like many residency programs, we hope our trainees will be future leaders in the field and in academia, so we place great importance on this, especially in more recent years now that quantified board scores are no longer a factor.” - Respondent #25
6	Research remains a high priority post-interview	Research is still a high priority.	At programs where research is highly valued and the goal is to produce academic neurosurgeons, research remains a significant factor in residency selection post-interview.	“I think it's still up there. Our program views itself as a very academic program. We want to train academic surgeons, and so a student's ability to be productive academically is an indicator that they are more likely to succeed in our program.” - Respondent #24
6	Factors other than research matter more post-interview	Research is a low priority compared to other components. An interview has an outsized impact	Some programs use research experience as part of an amalgamation of factors in selecting applicants for interviews. However, after the interview, other factors weigh more heavily in residency selection.	“I think this is interesting, but I would say that the research gets them through the front door, but the other stuff cements the deal. Again, when you look at people during, so, when you're doing the interview, you'll notice that some people have a lot of knowledge about their research experience, and some people have a lot of research on their application but don't really know it. And so, you have to tease through that. So, you know, I think most programs would value the intangibles that come from that in-person interview, the handshakes, the eye contact, the maturity that comes with handling conversation, the formation of career objectives and goals.” - Respondent #10
6	Research and other factors are given equal weighting post-interview	On equal footing with other components	At some programs, research is equally weighted with various other factors in determining who is selected for a residency spot.	“I think it's pretty equal. We had this last year, essentially, we were screening two, we had five sections, and one of those sections was research, so I would say it's on equal footing with those other four things, and that includes the personal statement, their ability to do clinical work, and sub internships and whatnot. The letter recommendations are in another part, and then the kind of extracurricular activities. It really is to us just on equal footing with those.” - Respondent #2
7	Research experiences are judged in light of opportunity	Denominator of opportunity for equity, excelling without opportunity gives a leg up; more opportunities should result in more productivity.	Research productivity is judged through the lens of the opportunities and resources an applicant had at their disposal. This applies both to students with a deficit in access and students with a surplus of access.	“We place a very high emphasis on the importance of their access, and that may have something to do with the fact that in Nebraska, we do have a neurosurgery program, but we also have a medical school without a neurosurgery training program…So, we sometimes become their adopted home program. And when I see students from those programs who have one or two, or let's call it relatively light CVs in terms of their scholarly activity, I absolutely take into account what resources they are trying to use…If you are at a powerhouse neuroscience, neurosurgery place, I expect you to have much more.” - Respondent #16
7	Differences in opportunities are not taken highly into consideration	Not highly considered.	Certain programs are seeking the most qualified candidates and believe that focusing on access to opportunities and resources is not a robust way to achieve this.	“This concept of 'distance traveled'—where they were and what they were able to do with that—is something we try to keep in mind. However, I'm not sure that for our program, we necessarily give someone with fewer resources a ton of slack because there are applicants out there every year that, despite fewer resources, are just as productive and competitive as others.” - Respondent #24
8	Current incentives lead to ethical issues, misguided decisions, and poor outcomes	Bad messaging leads students to select specialities too early, cultural emphasis on wrong metrics, a decrease in the quality of literature, ethical issues arising from the arms race, and forcing students to take time off.	Emphasizing unconstrained academic productivity may lead to a dysfunctional system. This can result in students feeling pressured to choose a specialty early to meet research requirements, which narrows their broader medical knowledge. It can also flood the literature with low-quality studies, exploit individuals to produce a high volume of research, and force students to take time off from medical school. This time off is biased towards affluent students, further exacerbating inequities.	“I think it's definitely a concern, and I think part of that concern stems from multiple things because you have some programs that I'm sure look at the number of publications and view that as a screen…There's a lot of self-interest here because a lot of faculty want medical students to do research because it helps them with their own research. So, you know, I think it's the type of thing that will eventually require some type of policy or statement that says let's put a cap on things before they get out of hand, because I think it's already starting to happen. And I think it's going to be a discussion, and I am very concerned about it. And I'm also concerned about the integrity of the system, because then if you just let it go, everyone does everything. It really erodes our system, but it's going to take debate.” - Respondent #4
8	The arms race is due to the difficulty of screening applicants	Difficulties of screening applicants	Due to the sheer number of applicants, program directors cannot conduct deep assessments of each applicant. As a result, certain metrics, such as board scores and research entries, are utilized to narrow the application pool.	“We don't have step one board scores as a marker; people are looking for other kinds of objective markers because you can't just scan through 300 applications, you have to figure out a way to cut down that number to make it more meaningful. And our concern is that, you know, the stack of research papers becomes the new board score and takes that number one spot. But we work very hard to fight against that.” - Respondent #11
8	Increasing research entries are not a concern	No issues with the arms race	Neurosurgery is a competitive field because there are more aspiring neurosurgeons than available residency slots. As a result, a "research arms race" occurs. This is a natural phenomenon and can be expected whenever there is high demand and low supply.	“There are limited spots for neurosurgery and quite a few candidates, and candidates have to find a way to. Achieve beyond the median of applicants, or else they will likely not match, so they have to be above average and be able to demonstrate that they are above average in some way that we can see in their application and interview process. And that is not unique to neurosurgery or unique to the 21st century…I think it's common to all fields involving high performers with limited positions and many candidates, so I think there's nothing bad about that other than that it's difficult for the applicants. But I mean, neurosurgery is very difficult. So I think that's reasonable.” - Respondent #26

Neurosurgery PDs appear to view research as one of several proxies for identifying attributes that suggest a medical student is well-prepared to succeed in residency. Research is often regarded as a way for students interested in neurosurgery to engage meaningfully with the field, demonstrate sustained commitment and dedication amid competing demands, and begin cultivating the habits and skills required in residency, where responsibilities related to patient care, self-directed learning, research, and personal life will compete for their time.

Quality of research was more frequently valued over quantity, with “quality” often defined as meaningful involvement in the research process. PDs reported assessing this through an applicant’s ability to articulate their research during interviews, as well as through sustained interest in a specific topic, evidenced by multiple publications within that area, particularly in high-impact journals.

When asked about the research “arms race,” PDs described increasing difficulty in screening applicants in the current landscape, which has intensified reliance on metrics such as research output. With the United States Medical Licensing Examination (USMLE) Step 1 now pass/fail, medical school grades often unreported, and hundreds of applicants competing for limited positions, distinguishing among candidates has become increasingly challenging. Many PDs expressed concern that this growing emphasis on research productivity fosters a system that prioritizes output over meaningful engagement or educational value, placing quantity above the quality of effort and learning.

Role of research in securing interviews and its weight post-interview

The majority of PDs emphasized that research is one of many factors considered when selecting applicants for interviews and typically plays a relatively minor role in that initial decision. However, several programs reported that prior research experience is a prerequisite for receiving an interview invitation, often citing alignment with their program’s research focus and institutional values. Notably, nearly all programs indicated that the weight placed on research diminishes significantly after the interview stage. Most PDs explicitly noted the disproportionately large influence of the interview itself on the final rank list, with only a few programs maintaining research as a significant consideration during the ranking process.

Categories of research valued by neurosurgery PDs and the influence of access on the evaluation of research output

When asked whether basic science, clinical, or other types of research are valued more highly, the majority of PDs indicated that no specific category is inherently favored. In a minority of responses, basic science research was described as potentially signaling a greater level of commitment and alignment with certain program values. However, these views appeared to reflect broader beliefs about the purpose of medical student research rather than a categorical preference for one type over another, as suggested by themes that emerged across multiple interview questions.

The majority of PDs reported considering an applicant’s access to research opportunities and institutional support when evaluating research output. Several PDs explicitly noted that students from well-resourced institutions may be judged more critically if they are not academically productive. As such, academic productivity is most often assessed within the context of available opportunities, with an emphasis on effort and engagement relative to access.

## Discussion

Around 81% of surveyed neurosurgery PDs expressed concern about the current state of applicant research, attributing the increased emphasis on research in part to the lack of objective metrics in the residency selection process. Research is broadly seen as a way for applicants to demonstrate genuine interest and commitment to neurosurgery while gaining meaningful experience. However, most PDs emphasized that the depth of inquiry and the ability to maximize available resources matter more than the sheer quantity or type of publications. With hundreds of applicants competing for a limited number of interview slots and even fewer residency positions, research is one of many factors considered in interview selection, but its influence diminishes significantly after the interview stage.

These findings contrast with observed trends in research output over the past decade. Wadhwa et al. reported that the average number of publications among matched neurosurgery interns was 1.7 ± 0.3 in 2008 and 5.5 ± 0.6 in 2018, with the median rising to 8 (IQR: 13) in the 2024 match [15]. Prior work has identified research output as a major source of anxiety for applicants. This pressure is increasingly evident: more students are attending research conferences and taking dedicated research years, and anecdotally, programs are receiving more inquiries from prospective applicants seeking research opportunities [16].

The neurosurgery research “arms race” has been a topic of growing concern and policy discussion. Suggested reforms have included limiting the number of research entries applicants can list on residency applications, restricting the number of programs to which they can apply, and developing specialty-specific application questions [17,18]. Recent publications have suggested the use of metrics aimed at scoring an applicant’s body of work with an emphasis on variables like first authorship or complex study design [19]. However, proposed changes remain contentious. Some argue such reforms could exacerbate inequities, particularly in the wake of the USMLE Step 1 transition to pass/fail, which may disproportionately disadvantage students from less prestigious institutions and international medical graduates (IMGs) [20]. A minority of our respondents viewed the increase in research output by residency candidates as beneficial for the field of neurosurgery, indicating that they did not view the current applicant research environment in a negative light.

Our findings offer a deeper understanding of this evolving landscape and have implications beyond neurosurgery for other competitive specialties in which research plays a central role. Notably, most PDs, including many from highly research-intensive institutions, reported that they do not prioritize publication count. Instead, they value the quality, thoughtfulness, and depth of research engagement, regardless of whether the work is basic science or clinical. Research was consistently described as one piece of a larger puzzle, important but secondary to factors like interview performance and program fit in final ranking decisions.

These findings point to two possible interpretations. Either PDs report these evaluative criteria but do not consistently apply them in practice, or they do apply them, and applicants have a limited understanding of how research is actually assessed. The latter may help explain the continued outsized emphasis on research quantity in medical student circles.

At first glance, the former interpretation might appear more convincing, especially given that the average number of research products (including abstracts, presentations, and publications) among matched neurosurgery applicants in 2024 was 37.4, with a median of eight publications (IQR: 13) [2]. However, this is not necessarily at odds with our findings and may reflect misunderstandings about how these numbers are reported and interpreted.

Gupta et al. analyzed the research output of 92.3% of matched neurosurgery applicants from 2017 to 2021 and confirmed that research output follows a right-skewed distribution. As such, they noted that medians and interquartile ranges are more appropriate descriptors than means and standard deviations. In their 2021 data, the median number of publications among the top 10% of applicants was 25.0 (IQR: 21.0-35.5), while the overall median among matched applicants was 5.0 (IQR: 2.0-11.0). The median number of first-author publications was 1.0 (IQR: 0.0-3.0), a figure that remained stable across all years analyzed. Gupta et al. also reported that approximately 50% of applicants had ≤5 publications, 25% had ≤2 publications, and that many successfully matched applicants had zero publications [21]. In contrast, the NRMP’s Charting Outcomes in the Match aggregates abstracts, presentations, and peer-reviewed publications into a single category approach that obscures these more nuanced differences and likely fuels applicant anxiety. These data highlight the distortion that can occur when research output is reported using averages, when multiple forms of scholarly activity are lumped together, and when attention is disproportionately focused on the small subset of applicants with the highest productivity.

In contrast to the belief that extreme research productivity is a prerequisite for matching into neurosurgery, the perspectives of PDs and recent data suggest that most programs adopt a more holistic view of research. The most practical takeaway is the need for both applicants and advisors to seek out more nuanced, data-informed perspectives. Reading interviews with PDs and examining studies that disaggregate research output can help clarify what matters most: meaningful, sustained engagement in research, not raw volume. This message is not only relevant for students but also for faculty and mentors guiding them. As findings such as these continue to challenge prevailing assumptions, they should increasingly inform the advising process.

For PDs, the challenge often lies in having the time and resources to evaluate the quality of applicant research amidst a flood of applications. Given this reality, a holistic review is aspirational but difficult to execute consistently. Tools like the Arms Race Control Score, currently being piloted through the Stop The Arms Race (STAR) study, may help programs estimate the quality and contextual relevance of applicant research more efficiently. As this work evolves, its potential to support fairer and more informed selection processes will be more thoroughly evaluated in the near future [19]. However, it is important to note that such scores may underrecognize some research contributions or may cause applicants to shift focus to work that is known to be higher-scoring as a new way of “gaming the system.” Individual programs may need to identify their own methods of scoring applicant research output based on their own values and priorities for residents. Ideally, they will provide transparency to applicants about these factors so that candidates have the opportunity to determine programs that will be the right fit for their academic aspirations.

Limitations and strengths

While our study had a 26.3% response rate, which introduces the possibility of non-response bias, all neurosurgery PDs in the United States were contacted via email using a standardized and uniform recruitment strategy to minimize selection bias. The participating programs demonstrated meaningful variation in geographic region, resident class size, and NIH funding status, as shown in Table 1.

Differences in opinion among PDs did not appear to correlate with NIH funding level, geographic location, program size, or any other identifiable institutional factor. For example, even among directors from programs with the highest levels of NIH funding, perspectives on key topics varied significantly. This suggests that individual program culture or the personal views of the PD interviewed are likely the primary driver of differing opinions.

It remains possible that PDs with particularly strong views on research were more likely to participate. Nonetheless, the interviews revealed a broad range of perspectives across nearly all key questions. While general agreement emerged on several issues, notable disagreement also surfaced, most commonly along lines of “research-focused” versus “less research-focused” programs. This mix of consensus and divergence was particularly evident in responses concerning the perceived research “arms race.”

Structured interviews were intentionally used to ensure consistency across participants, especially given that interviewers were medical students without formal qualitative training. To preserve standardization and accommodate the time constraints of participants, follow-up probing was deliberately limited. As a result, interview durations were relatively short for qualitative inquiry, with a mean length of thirteen minutes. The relatively short average interview length may have limited the depth and richness of responses. 

Hawthorne bias may have also influenced responses, as participants were aware they were being interviewed and recorded. Verbal consent was obtained prior to each interview, during which participants were informed that recordings would remain confidential and be stored on a password-protected device accessible only to the research team. Interviewers also emphasized that participants could skip questions or withdraw at any time, which may have helped reduce pressure to conform to perceived expectations.

The absence of member checking may limit confirmation that themes fully reflect participants’ intended meanings, potentially impacting the credibility of findings. However, this limitation was addressed through independent coding, adjudicated resolution of discrepancies, and consensus-based theme development. Investigator triangulation further supported the trustworthiness of the analysis. Additionally, because the research team included medical students pursuing careers in neurosurgery, introducing potential bias in data collection and interpretation, we incorporated reflexive practices such as regular team discussions and critical awareness of positionality to mitigate bias and ensure balanced theme development.

## Conclusions

The residency selection process is undergoing a significant transformation, especially with the shift of USMLE Step 1 to a pass-fail system. Our study reveals that neurosurgery PDs generally view research as a critical indicator of an applicant's potential impact and dedication. They evaluate research based on depth of inquiry, journal quality, publication quantity, authorship position, and interview discussions, favoring high-quality research. PDs also consider disparities in research resources, evaluating productivity within the context of institutional support. However, our findings also reveal that there remain varied perspectives about the concept of an “arms race” and whether this is deleterious or simply a neutral feature of a competitive field.

Proposed policy changes, such as capping research entries on applications or creating a specialty-specific application, may offset the emphasis on research and provide a more comprehensive, easily digestible view of applicants. However, concerns exist that these changes might disadvantage students from less prestigious institutions and IMGs, who often rely on research output to demonstrate their capabilities and determination. Our study revealed that there is no clear consensus amongst PDs about the need for or utility of these potential modifications to the application. Given this variability, increased transparency from programs to applicants about their specific values and priorities may help reduce applicant anxiety and help applicants focus on programs that are the best fit for their academic background and aspirations.

## Appendices

Appendix 1

Question 1: Aspiring neurosurgeons devote a significant amount of time and effort to generating a substantial volume of publications in pursuit of matching. In light of this, what is your program's perspective on the educational purpose of medical student research in the field?

Question 2: In general, what factors do you use to determine the research quality of an applicant’s publications?

Question 3: Do you believe there is a conflict between quantity and quality, and if so, which one does your program favor and why?

Question 4: As you are likely aware, biomedical research may exist anywhere on a spectrum from foundational or basic science, such as the identification of novel genes or biological mechanisms, through clinical investigation focused on individual patient or population-level outcomes. Considering this, does your program place greater value on either clinical or basic science research experience, and why?

Question 5: There are numerous components considered in the residency application process, including academic metrics (i.e., board scores, grades, class rank), extracurricular involvement and leadership, letters of recommendation, personality characteristics, and abilities observed during sub-internships. How does your program perceive the significance of an applicant’s research experience when determining who is selected for an interview?

Question 6: After the interview, to what extent does an applicant's research experience influence the final ranking for the match compared to other components of the applicant's profile?

Question 7: How does your program take into account an applicant's access to resources and opportunities, such as access to research support staff and infrastructure, the presence of a home academic neurosurgery program, and/or the ability to dedicate time for research when evaluating their research experiences and productivity?

Question 8: According to NRMP outcomes data, there is a temporal trend towards higher board scores and more numerous research experiences in individuals successfully matching into competitive specialties, such as neurosurgery. This phenomenon has been termed an arms race by several leaders in medical education. Do you have concerns about how this trend might be impacting the residency application process and/or the quality of neurosurgical investigation? If so, would you mind elaborating on what your concerns are and why?
